# Association between alcohol consumption and kidney stones in American adults: 2007–2016 NHANES

**DOI:** 10.3389/fpubh.2023.1156097

**Published:** 2023-04-14

**Authors:** Zhen Zhou, Zhicong Huang, Guoyao Ai, Xin Guo, Guohua Zeng, Wei Zhu

**Affiliations:** Guangdong Key Laboratory of Urology and Department of Urology, The First Affiliated Hospital of Guangzhou Medical University, Guangzhou, Guangdong, China

**Keywords:** kidney stones, alcohol consumption, NHANES, association, cross-sectional analysis

## Abstract

**Purpose:**

To investigate the association between alcohol consumption and kidney stones in American adults.

**Materials and methods:**

National Health and Nutrition Examination Survey (NHANES) datasets from 2007 to 2016 were utilized. Participants with a history of kidney stones and alcohol consumption aged 20 or older were included. Weighted proportions and regression analysis were used to assess the association between alcohol consumption and kidney stones by adjusting age, gender, race, marital status, education, recreational activities, smoking, and several comorbidities.

**Results:**

Eleven population samples (Q1-Q11) were included from the NHANES dataset based on 11 questions compiled from the Alcohol Use Questionnaire (ALQ). In the fully adjusted regression model, none of these 11 samples demonstrated a significant association with urolithiasis, that is, alcohol consumption was not significantly associated with the incidence of kidney stones, even among heavy drinkers.

**Conclusion:**

Alcohol consumption is not significantly associated with the prevalence of kidney stones. This finding requires a more adequate sample size and a more detailed review of the history of kidney stones to be further verified.

## Introduction

Kidney stones have a high incidence rate worldwide that has been increasing every year (1–4). Alcohol is a common addictive substance, with about a third of the world’s population currently consuming it for different reasons (5). Previous studies have shown that fluid intake may be associated with kidney stone formation (6, 7), and alcohol is one of the important liquids. Some researchers believe that alcohol intake is negatively associated with the formation of kidney stones (8–12), while others believe that alcohol consumption is a risk factor for kidney stone formation (13, 14). We believe that research on the relationship between alcohol consumption and kidney stones is necessary to generate new ideas for the prevention and treatment of kidney stones, given the differing opinions.

The purpose of this study was to investigate the relationship between different drinking profiles and history of kidney stones by analyzing National Health and Nutrition Examination Survey (NHANES) cross-sectional data.

## Materials and methods

### Data resource and participants

**Figure 1:**
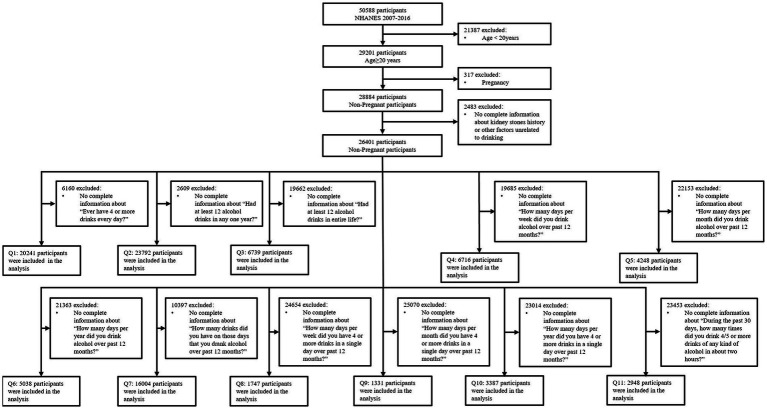
Flow chart of the study population.

NHANES, a major program of the National Center for Health Statistics (NCHS), is a program of studies designed to estimate the health and nutritional status of participants in the United States. The survey combines interviews and physical examinations. We utilized 5 continuous cycles of NHANES data from year 2007 to 2016. This cross-sectional study included 29,201 adult American participants aged 20 years or older. 317 subsequently pregnant participants were excluded. Furthermore, 2,483 participants with incomplete kidney stones history information or other factors unrelated to drinking were excluded. Finally, we compiled 11 questions from the Alcohol Use Questionnaire (ALQ), and excluded participants with incomplete information for each question separately to obtain 11 different samples, named Q1 to Q11. Detailed inclusion and exclusion standards are shown in Figure 1. Additionally, there were minor variations observed in the ALQs of certain cycles in NHANES. Therefore, the questions used to collect specific samples (Q1, Q4, Q5, Q6, Q8, Q9, Q10) underwent processing, as described in Supplementary Figure S1.

### Results and exposure factors

Results of the questionnaire determines whether the participant has a history of nephrolithiasis. If the answer to the question “Have you ever had kidney stones?” is “yes,” we assume that the participant has a history of kidney stones. The veracity of self-reported was confirmed in a previous study (15).

The main exposure factor was the lifetime and current (during the past 12 months) alcohol consumption of the participants, as determined by the ALQ in NHANES 2007–2016. The type of alcohol consumed was not specified.

### Covariates

In order to increase accuracy and credibility, we included the following covariates: age, gender, race, marital status, education, recreational activities, smoking, asthma, overweight, gout, congestive heart failure, coronary heart disease, angina, stroke, cancer, diabetes, hypertension, and BMI. According to the recommendations of NHANES, we devided the age into three groups: 20–39, 40–59, and ≥ 60 years. Races included Mexican American, other Hispanic, non-Hispanic white, non-Hispanic black, and other races. Marital status was categorized as married, widowed, divorced, separated, never married, and living with partner. Education level included less than 9th grade, 9-11th grade (includes 12th grade with no diploma), high school graduate/GED or equivalent, some college or AA degree, and college graduate or above. BMI was divided into <25, 25–30, and > 30 kg/m^2^. The comorbidities included asthma, overweight, gout, congestive heart failure, coronary heart disease, angina, stroke, cancer, diabetes, and hypertension.

### Statistical methods

We used the NHANES recommended weighting data and the merging method. In the baseline characteristics table, categorical variables are expressed as proportions, and all continuous variables are treated as categorical variables, also expressed as proportions. The difference between those with and without kidney stones history was tested by survey-weighted Chi-square test.

To investigate the relationship between alcohol consumption and kidney stones prevalence, we applied three logistic regression models, including unadjusted, slightly adjusted, and fully adjusted covariates. Crude model was unadjusted. Model I was adjusted for age, gender and race. Model II was adjusted for age, gender, race, marital status, education, recreational activities, smoking, asthma, overweight, gout, congestive heart failure, coronary heart disease, angina, stroke, cancer, diabetes, hypertension, and BMI. The same method was used to examine 11 different population samples separately to explore the association between alcohol consumption and kidney stones prevalence. To better evaluate the relationship, we treated the continuous variables in the 11 samples as categorical variables and analyzed them to estimate trends. To examine the association between heavy drinking and kidney stones, we classified Q11 as <5 and ≥ 5, based on the Substance Abuse and Mental Health Services Administration (SAMHSA) definitions of heavy drinking and binge drinking, respectively. Additionally, we conducted a separate analysis to explore the relationship between the number of binge drinking days and kidney stones, using Q11 as a continuous variable. Finally, we performed a univariate analysis of the association of all confounders listed in the baseline tables with kidney stones.

We merged the sample weights of 5 continuous cycles according to the recommended method on the NHANES website.^1^ According to the suggestions, we used a weight that was appropriate for the variable of interest that was collected on the smallest number of respondents. All analyses were performed using R packages (http://www.R-project.org; The R Foundation) and EmpowerStats (www.empowerstats.com, X&Y solutions Inc., Boston, MA.). A 2-tailed *p* < 0.05 was considered statistically significant.

## Ethics statement

The studies involving human participants were reviewed and approved by National Center for Health Statistics (NCHS) research ethics review board. The patients/participants provided their written informed consent to participate in this study

## Results

### Participants characteristics

Figure 1 shows the study design, inclusion and exclusion criteria. Eleven groups of American adults were included in the corresponding sample (Q1-Q11), and the number of people in each group is also shown. Supplementary Figure S1 shows the process of some questions in the ALQs of certain cycles in NHANES for better accessibility to the study sample (Q1, Q4, Q5, Q6, Q8, Q9, Q10). Table 1 shows the baseline demographic characteristics of the participants based on kidney stones history, using the population prior to classification by the ALQ. Patients with a history of kidney stones may be more likely to have other comorbidities than those without a history of kidney stones. Different histories of kidney stones may not be related to the level of education.

**Table 1:** Baseline characteristics of participants with and without kidney stone history.

	Without history		With history		*p* value^b^
	Non-weighted NO.	% (95% CI)^a^	Non-weighted NO.	% (95% CI)^a^	
Total number of participants	23,986		2,415		
Age					<0.0001
20–39	8,532	38.46 (36.99,39.94)	467	21.36 (19.32,23.56)	
40–59	8,025	37.37 (36.37,38.38)	834	42.49 (39.41,45.63)	
≥60(%)	7,429	24.18 (23.12,25.26)	1,114	36.14 (33.56,38.81)	
Gender					<0.0001
Male	11,559	47.85 (47.18,48.52)	1,338	54.78 (52.20,57.34)	
Famale	12,427	52.15 (51.48,52.82)	1,077	45.22 (42.66,47.80)	
Race					<0.0001
Mexican American	3,684	8.72 (7.18,10.55)	320	6.08 (4.53,8.10)	
Other Hispanic	2,543	5.78 (4.76,7.00)	286	5.19 (3.82,7.01)	
Non-Hispanic White	9,719	65.71 (62.46,68.83)	1,306	77.33 (73.79,80.52)	
Non-Hispanic Black	5,290	11.90 (10.33,13.68)	321	5.92 (4.84,7.22)	
Other Race - Including Multi-Racial	2,750	7.89 (6.96,8.92)	182	5.48 (4.28,7.00)	
Marital status					<0.0001
Married	12,134	54.28 (52.77,55.78)	1,406	63.45 (60.56,66.24)	
Widowed	1825	5.61 (5.23,6.02)	243	6.54 (5.67,7.53)	
Divorced	2,544	10.00 (9.43,10.60)	315	12.35 (10.77,14.13)	
Separated	806	2.39 (2.13,2.67)	85	2.23 (1.63,3.04)	
Never married	4,733	19.64 (18.24,21.12)	223	9.58 (8.14,11.25)	
Living with partner	1944	8.09 (7.52,8.70)	143	5.85 (4.75,7.19)	
Education level					0.262
Less than 9th grade	2,600	5.80 (5.19,6.47)	277	5.48 (4.54,6.60)	
9-11th grade (Includes 12th grade with no diploma)	3,492	11.05 (10.08,12.11)	354	11.29 (9.82,12.94)	
High school graduate/GED or equivalent	5,426	22.03 (20.99,23.11)	549	23.23 (20.61,26.07)	
Some college or AA degree	6,895	31.26 (30.21,32.34)	733	32.65 (30.25,35.14)	
College graduate or above	5,573	29.86 (27.91,31.87)	502	27.35 (23.81,31.21)	
Moderate or vigorous recreational activities at least 10 min continuously					<0.0001
NO	12,423	45.43 (43.64,47.24)	1,453	53.62 (50.74,56.48)	
YES	11,563	54.57 (52.76,56.36)	962	46.38 (43.52,49.26)	
Smoked at least 100 cigarettes in life					0.0001
NO	13,523	56.14 (54.89,57.38)	1,194	50.20 (47.05,53.35)	
YES	10,463	43.86 (42.62,45.11)	1,221	49.80 (46.65,52.95)	
Asthma					0.0004
NO	20,654	85.55 (84.85,86.22)	2,000	82.45 (80.45,84.29)	
YES	3,332	14.45 (13.78,15.15)	415	17.55 (15.71,19.55)	
Overweight					<0.0001
NO	16,137	67.00 (66.05,67.94)	1,324	52.84 (49.93,55.73)	
YES	7,849	33.00 (32.06,33.95)	1,091	47.16 (44.27,50.07)	
Gout					<0.0001
NO	23,026	96.66 (96.27,97.01)	2,207	91.84 (90.33,93.13)	
YES	960	3.34 (2.99,3.73)	208	8.16 (6.87,9.67)	
Congestive heart failure					<0.0001
NO	23,341	97.98 (97.71,98.23)	2,261	95.29 (94.26,96.15)	
YES	645	2.02 (1.77,2.29)	154	4.71 (3.85,5.74)	
Coronary heart disease					<0.0001
NO	23,178	97.23 (96.89,97.54)	2,221	93.30 (91.98,94.42)	
YES	808	2.77 (2.46,3.11)	194	6.70 (5.58,8.02)	
Angina					<0.0001
NO	23,503	98.33 (98.08,98.54)	2,287	95.48 (94.53,96.28)	
YES	483	1.67 (1.46,1.92)	128	4.52 (3.72,5.47)	
Stroke					<0.0001
NO	23,191	97.51 (97.23,97.76)	2,279	95.58 (94.80,96.24)	
YES	795	2.49 (2.24,2.77)	136	4.42 (3.76,5.20)	
Cancer					<0.0001
NO	21,873	90.56 (90.02,91.07)	2,050	84.02 (82.22,85.67)	
YES	2,113	9.44 (8.93,9.98)	365	15.98 (14.33,17.78)	
Diabetes					<0.0001
NO	21,124	91.34 (90.72,91.92)	1,865	81.71 (80.01,83.30)	
YES	2,862	8.66 (8.08,9.28)	550	18.29 (16.70,19.99)	
Hypertension					<0.0001
NO	15,878	70.29 (69.21,71.35)	1,209	54.51 (51.91,57.08)	
YES	8,108	29.71 (28.65,30.79)	1,206	45.49 (42.92,48.09)	
BMI					<0.0001
<25	7,345	31.73 (30.53,32.96)	496	20.58 (18.39,22.96)	
25–30	7,977	33.61 (32.70,34.54)	837	33.41 (31.12,35.78)	
>30	8,664	34.65 (33.54,35.78)	1,082	46.01 (43.69,48.36)	

a % (95% CI), survey-weighted percentage (95% CI).

b value of *p* was by survey-weighted Chi-square test.

### Regression analysis

We put 11 samples (Q1-Q11) related to alcohol consumption into three logical regression models for analysis. Although Q1 was negatively correlated with the risk of kidney stones in crude model (OR = 0.70; 95%CI 0.60 to 0.82) and model I (OR = 0.76; 95%CI 0.64 to 0.90), it was not associated with kidney stones in model II (OR = 0.86; 95%CI 0.72 to 1.03). Q4 was positively correlated with the risk of kidney stones in crude model (OR = 1.34; 95%CI 1.02 to 1.76), but not in model I (OR = 1.01; 95%CI 0.76 to 1.33) and model II (OR = 1.06; 95%CI 0.79 to 1.40). The remaining samples were not associated with kidney stones in all three logical regression models (Table 2). In addition, we individually analyzed the effect of each covariate on the risk of kidney stones. When confounders were not considered, women appeared to be less likely to have kidney stones than men (OR = 0.76; 95% CI 0.68 to 0.85), and the risk of kidney stones seemed to be higher in those who smoked, were obese, and had other medical conditions. Interestingly, if covariates were not considered, those who were never married appeared to have a lower risk of kidney stones than those who were married (OR = 0.42; 95% CI 0.34 to 0.51). Furthermore, there was no association between education level and the risk of kidney stones (Table 3).

**Table 2:** Association between alcohol consumption and kidney stone.

Exposure	Crude Model^a^		Model I^b^		Model II^c^	
	OR (95%CI)	value of *p*	OR (95%CI)	*p*-value	OR(95%CI)	*p*-value
Q1						
NO	Ref.		Ref.		Ref.	
YES	0.70 (0.60, 0.82)	<0.0001	0.76 (0.64, 0.90)	0.0023	0.86 (0.72, 1.03)	0.1128
Q2						
NO	Ref.		Ref.		Ref.	
YES	1.07 (0.94, 1.22)	0.2862	1.13 (0.98, 1.30)	0.089	1.14 (0.99, 1.32)	0.0716
Q3						
NO	Ref.		Ref.		Ref.	
YES	0.91 (0.71, 1.16)	0.4441	0.94 (0.73, 1.22)	0.6616	1.02 (0.79, 1.31)	0.8703
Q4						
1–3	Ref.		Ref.		Ref.	
4–7	1.34 (1.02, 1.76)	0.039	1.01 (0.76, 1.33)	0.9633	1.06 (0.79, 1.40)	0.7115
Q5						
1–10	Ref.		Ref.		Ref.	
11–20	1.48 (0.62, 3.52)	0.3803	1.32 (0.55, 3.16)	0.5388	1.47 (0.63, 3.45)	0.3766
21–30	0.49 (0.12, 1.99)	0.3229	0.32 (0.08, 1.32)	0.1194	0.43 (0.10, 1.79)	0.2512
P-value for trend	0.9079		0.5987		0.9721	
Q6						
1–120	Ref.		Ref.		Ref.	
121–240	1.95 (0.61, 6.22)	0.2607	1.51 (0.48, 4.73)	0.4811	1.59 (0.51, 4.95)	0.4263
241–365	0.11 (0.02, 0.53)	0.0072	0.08 (0.02, 0.38)	0.0023	0.13 (0.03, 0.60)	0.0126
P-value for trend	0.5342		0.2073		0.4758	
Q7						
1–15	Ref.		Ref.		Ref.	
≥16	0.48 (0.15, 1.54)	0.2232	0.64 (0.20, 2.03)	0.4507	0.63 (0.19, 2.09)	0.4565
Q8						
1–3	Ref.		Ref.		Ref.	
4–7	1.18 (0.72, 1.95)	0.5113	0.89 (0.49, 1.62)	0.703	0.80 (0.43, 1.49)	0.4875
Q9						
1–10	Ref.		Ref.		Ref.	
11–20	0.55 (0.07, 4.25)	0.5677	0.57 (0.07, 4.64)	0.6001	0.64 (0.08, 5.44)	0.6858
21–30	0.00 (0.00, 0.00)	<0.0001	0.00 (0.00, 0.00)	<0.0001	0.00 (0.00, 0.00)	<0.0001
*p*-value for trend	0.3672		0.4079		0.5611	
Q10						
1–120	Ref.		Ref.		Ref.	
121–240	4.98 (1.06, 23.39)	0.0452	5.09 (1.07, 24.11)	0.0442	5.23 (1.24, 22.15)	0.0292
241–365	0.00 (0.00, 0.00)	<0.0001	0.00 (0.00, 0.00)	<0.0001	0.00 (0.00, 0.00)	<0.0001
*p*-value for trend	0.4208		0.5468		0.5108	
Q11(Continuous)	0.95 (0.85, 1.06)	0.3381	0.97 (0.89, 1.07)	0.5559	0.98 (0.90, 1.07)	0.7434
<5	Ref.		Ref.		Ref.	
≥5	0.72 (0.25, 2.02)	0.5338	0.85 (0.30 2.39)	0.7588	0.88 (0.31, 2.53)	0.8497

a Crude Model: adjusted for none.

b Model I: adjusted for age, gender and race.

c Model II: adjusted for age, gender, race, marital status, education, recreational activities, smoking, asthma, overweight, gout, congestive heart failure, coronary heart disease, angina, stroke, cancer, diabetes, hypertension, and BMI.

**Table 3:** Univariate analysis for kidney stone.

Covariate	(N^a^) % (95%CI)^b^	OR (95%CI)	*p*-value^c^
Age(years)	(26401) 9.50 (8.98,10.01)	1.02 (1.02, 1.03)	<0.0001
20–39	(8999) 5.51 (4.89,6.13)	Ref.	
40–59	(8859) 10.66 (9.67,11.65)	2.05 (1.75, 2.40)	<0.0001
≥60	(8543) 13.56 (12.57,14.55)	2.69 (2.35, 3.08)	<0.0001
Gender			
Male	(12897) 10.72 (9.99,11.46)	Ref.	
Famale	(13504) 8.34 (7.66,9.02)	0.76 (0.68, 0.85)	<0.0001
Race			
Mexican American	(4004) 6.82 (5.90,7.73)	Ref.	
Other Hispanic	(2829) 8.61 (7.26,9.97)	1.29 (1.02, 1.63)	0.0356
Non-Hispanic White	(11025) 10.99 (10.29,11.69)	1.69 (1.44, 1.98)	<0.0001
Non-Hispanic Black	(5611) 4.96 (4.40,5.51)	0.71 (0.59, 0.86)	0.0009
Other Race - Including Multi-Racial	(2932) 6.80 (5.43,8.17)	1.00 (0.76, 1.32)	0.9857
Marital status			
Married	(13540) 10.93 (10.13,11.72)	Ref.	
Widowed	(2068) 10.89 (9.59,12.19)	1.00 (0.84, 1.18)	0.9652
Divorced	(2859) 11.48 (9.85,13.10)	1.06 (0.89, 1.26)	0.532
Separated	(891) 8.92 (6.38,11.47)	0.80 (0.59, 1.09)	0.1569
Never married	(4956) 4.87 (4.11,5.63)	0.42 (0.34, 0.51)	<0.0001
Living with partner	(2087) 7.06 (5.69,8.42)	0.62 (0.49, 0.78)	0.0001
Education level			
Less than 9th grade	(2877) 9.03 (7.68,10.37)	Ref.	
9-11th grade (Includes 12th grade with no diploma)	(3846) 9.68 (8.55,10.81)	1.08 (0.87, 1.33)	0.4767
High school graduate/GED or equivalent	(5975) 9.96 (8.80,11.13)	1.12 (0.92, 1.36)	0.2774
Some college or AA degree	(7628) 9.88 (9.06,10.69)	1.10 (0.93, 1.31)	0.2622
College graduate or above	(6075) 8.77 (7.80,9.74)	0.97 (0.78, 1.21)	0.7791
Moderate or vigorous recreational activities at least 10 min continuously			
NO	(13876) 11.02 (10.34,11.70)	Ref.	
YES	(12525) 8.19 (7.55,8.82)	0.72 (0.65, 0.80)	<0.0001
Smoked at least 100 cigarettes in life			
NO	(14717) 8.58 (8.02,9.14)	Ref.	
YES	(11684) 10.65 (9.74,11.55)	1.27 (1.13, 1.43)	0.0002
Asthma			
NO	(22654) 9.19 (8.66,9.71)	Ref.	
YES	(3747) 11.30 (10.06,12.54)	1.26 (1.11, 1.43)	0.0008
Overweight			
NO	(17461) 7.64 (7.07,8.21)	Ref.	
YES	(8940) 13.04 (12.06,14.02)	1.81 (1.61, 2.04)	<0.0001
Gout			
NO	(25233) 9.07 (8.55,9.58)	Ref.	
YES	(1168) 20.40 (17.03,23.76)	2.57 (2.06, 3.20)	<0.0001
Congestive heart failure			
NO	(25602) 9.26 (8.75,9.77)	Ref.	
YES	(799) 19.66 (15.64,23.69)	2.40 (1.84, 3.12)	<0.0001
Coronary heart disease			
NO	(25399) 9.15 (8.62,9.68)	Ref.	
YES	(1002) 20.26 (16.89,23.63)	2.52 (2.01, 3.17)	<0.0001
Angina			
NO	(25790) 9.25 (8.73,9.77)	Ref.	
YES	(611) 22.05 (18.12,25.99)	2.78 (2.18, 3.54)	<0.0001
Stroke			
NO	(25470) 9.33 (8.80,9.85)	Ref.	
YES	(931) 15.69 (13.01,18.37)	1.81 (1.46, 2.24)	<0.0001
Cancer			
NO	(23923) 8.87 (8.33,9.41)	Ref.	
YES	(2478) 15.08 (13.53,16.64)	1.82 (1.59, 2.10)	<0.0001
Diabetes			
NO	(22989) 8.58 (8.06,9.11)	Ref.	
YES	(3412) 18.14 (16.43,19.85)	2.36 (2.07, 2.69)	<0.0001
Hypertension			
NO	(17087) 7.53 (6.98,8.07)	Ref.	
YES	(9314) 13.84 (12.86,14.83)	1.97 (1.76, 2.21)	<0.0001
BMI(kg/m^2^)	(26401) 9.50 (8.98,10.01)	1.04 (1.03, 1.04)	<0.0001
<25	(7841) 6.37 (5.63,7.11)	Ref.	
25–30	(8814) 9.44 (8.66,10.23)	1.53 (1.31, 1.80)	<0.0001
>30	(9746) 12.23 (11.33,13.13)	2.05 (1.78, 2.36)	<0.0001

CI, confidence interval. OR, odds ratio.

a N: Number of observed.

b % (95%CI): survey-weighted percentage (95% CI).

c For Kidney stone: survey-weighted OR (95%CI) *p*-value.

## Discussion

This cross-sectional study investigated the association between alcohol consumption and history of kidney stones by analyzing 5 cycles of the NHANES dataset. The results displayed that there was no significant correlation between both lifetime and current (past 12 months) alcohol consumption and a history of kidney stones, that is, there was no significant association between the amount and frequency of alcohol consumption and the prevalence of kidney stones, even among heavy drinkers.

Consuming alcohol is a mean of socializing in many cultures. Unfortunately, of the population that do consume alcohol, many is addicted and drink it without restraint. Alcohol consumption is associated with a series of health problems, such as diabetes, obesity (16, 17) etc. Diabetes and obesity have previously been reported to be risk factors for kidney stone formation (18, 19). Furthermore, alcohol consumption can cause kidney injury, inflammation and fibrosis, all of which are associated with the formation of kidney stones (20–22). Nevertheless, few studies have elucidated the direct correlation between alcohol consumption and kidney stones. In this study, although the direct association between alcohol consumption and kidney stones does not appear to be significant, other diseases associated with kidney stone formation due to alcohol consumption should be considered.

This study poses some limitations. First, we noticed that some questions in the ALQ explored alcohol consumption of participants in the last 12 months, however, the specific times of kidney stone occurrence for the corresponding participants were not specified in NHANES. As a result, it is unclear how data of participants with kidney stones that occurred before the last 12 months affect the results of this study. In addition, the ALQ questionnaire did not specify the type of alcohol consumed, which limits our ability to assess whether there is an association between different types of alcohol or alcoholic beverages and the formation of kidney stones. Furthermore, detailed data on the composition, size, and treatment history of kidney stones were not available in NHANES, therefore limiting our ability to investigate the correlation between alcohol consumption and different types of kidney stones.

## Conclusion

By conducting a cross-sectional study of the available data in the NHANES dataset, we found that alcohol consumption is not significantly associated with the prevalence of kidney stones. This finding requires a more adequate sample size and a more detailed review of the history of kidney stones to be further verified.

## Data availability statement

Publicly available datasets were analyzed in this study. This data can be found here: NHANES (https://www.cdc.gov/nchs/nhanes/index.htm).

## Ethics statement

The studies involving human participants were reviewed and approved by National Center for Health Statistics (NCHS) research ethics review board. The patients/participants provided their written informed consent to participate in this study.

## Author contributions

ZZ and WZ contributed to conception and design of the study. ZZ organized the database. ZZ, ZH, GA, and GZ participated in acquisition of data. ZZ performed the statistical analysis. ZZ wrote the first draft of the manuscript. XG contributed to manuscript revision. All authors contributed to the article and approved the submitted version.

## Funding

This work was granted and financed by the Young Talent Support Project of Guangzhou Association for Science and Technology, and the Guangzhou Science Technology and Innovation Commission (no. 202102010214).

## Conflict of interest

The authors declare that the research was conducted in the absence of any commercial or financial relationships that could be construed as a potential conflict of interest.

## Publisher’s note

All claims expressed in this article are solely those of the authors and do not necessarily represent those of their affiliated organizations, or those of the publisher, the editors and the reviewers. Any product that may be evaluated in this article, or claim that may be made by its manufacturer, is not guaranteed or endorsed by the publisher.

## Footnotes

^1^https://www.cdc.gov/nchs/nhanes/index.htm

## References

[1] Soucie JM Thun MJ Coates RJ McClellan W Austin H. Demographic and geographic variability of kidney stones in the United States. Kidney Int. (1994) 46:893–9. doi: 10.1038/ki.1994.347, PMID: 7996811

[2] Pozdzik A Maalouf N Letavernier E Brocheriou I Body JJ Vervaet B. Meeting report of the "symposium on kidney stones and mineral metabolism: calcium kidney stones in 2017". J Nephrol. (2019) 32:681–98. doi: 10.1007/s40620-019-00587-1, PMID: 30680550

[3] Scales CJ Smith AC Hanley JM Saigal CS. Prevalence of kidney stones in the United States. Eur Urol. (2012) 62:160–5. doi: 10.1016/j.eururo.2012.03.052, PMID: 22498635 PMC3362665

[4] Croppi E Ferraro PM Taddei L Gambaro G. Prevalence of renal stones in an Italian urban population: a general practice-based study. Urol Res. (2012) 40:517–22. doi: 10.1007/s00240-012-0477-z 22534684

[5] Ilhan MN Yapar D. Alcohol consumption and alcohol policy. Turk J Med Sci. (2020) 50:1197–202. doi: 10.3906/sag-2002-237, PMID: 32421277 PMC7491269

[6] Gamage KN Jamnadass E Sulaiman SK Pietropaolo A Aboumarzouk O Somani BK. The role of fluid intake in the prevention of kidney stone disease: a systematic review over the last two decades. Turk J Urol. (2020) 46:S92–S103. doi: 10.5152/tud.2020.20155, PMID: 32525478 PMC7731957

[7] Cheungpasitporn W Rossetti S Friend K Erickson SB Lieske JC. Treatment effect, adherence, and safety of high fluid intake for the prevention of incident and recurrent kidney stones: a systematic review and meta-analysis. J Nephrol. (2016) 29:211–9. doi: 10.1007/s40620-015-0210-4, PMID: 26022722 PMC4831051

[8] Turney BW Appleby PN Reynard JM Noble JG Key TJ Allen NE. Diet and risk of kidney stones in the Oxford cohort of the European prospective investigation into cancer and nutrition (EPIC). Eur J Epidemiol. (2014) 29:363–9. doi: 10.1007/s10654-014-9904-5, PMID: 24752465

[9] Littlejohns TJ Neal NL Bradbury KE Heers H Allen NE Turney BW. Fluid intake and dietary factors and the risk of incident kidney stones in UK biobank: a population-based prospective cohort study. Eur Urol Focus. (2020) 6:752–61. doi: 10.1016/j.euf.2019.05.002, PMID: 31085062

[10] Hirvonen T Pietinen P Virtanen M Albanes D Virtamo J. Nutrient intake and use of beverages and the risk of kidney stones among male smokers. Am J Epidemiol. (1999) 150:187–94. doi: 10.1093/oxfordjournals.aje.a009979, PMID: 10412964

[11] de Lorimier AA. Alcohol, wine, and health. Am J Surg. (2000) 180:357–61. doi: 10.1016/S0002-9610(00)00486-4 11137687

[12] Wang H Fan J Yu C Guo Y Pei P Yang L. Consumption of tea, alcohol, and fruits and risk of kidney stones: a prospective cohort study in 0.5 million Chinese adults. Nutrients. (2021) 13:1119. doi: 10.3390/nu13041119 33805392 PMC8065818

[13] Lee YH Huang WC Tsai JY Lu CM Chen WC Lee MH. Epidemiological studies on the prevalence of upper urinary calculi in Taiwan. Urol Int. (2002) 68:172–7. doi: 10.1159/000048445, PMID: 11919463

[14] Khalili P Jamali Z Sadeghi T Esmaeili-Nadimi A Mohamadi M Moghadam-Ahmadi A. Risk factors of kidney stone disease: a cross-sectional study in the southeast of Iran. BMC Urol. (2021) 21:141. doi: 10.1186/s12894-021-00905-5. 34625088 PMC8499392

[15] Taylor EN Stampfer MJ Curhan GC. Obesity, weight gain, and the risk of kidney stones. JAMA. (2005) 293:455–62. doi: 10.1001/jama.293.4.455, PMID: 15671430

[16] Liu M Park S. A causal relationship between alcohol intake and type 2 diabetes mellitus: a two-sample Mendelian randomization study. Nutr Metab Cardiovasc Dis. (2022) 32:2865–76. doi: 10.1016/j.numecd.2022.08.013, PMID: 36184363

[17] Traversy G Chaput JP. Alcohol consumption and obesity: an update. Curr Obes Rep. (2015) 4:122–30. doi: 10.1007/s13679-014-0129-4, PMID: 25741455 PMC4338356

[18] Song L Maalouf NM. Nephrolithiasis. South Dartmouth (MA): MDText.com, Inc (2020). 2000 p.

[19] Carbone A Al SY Tasca A Palleschi G Fuschi A Nunzio CD. Obesity and kidney stone disease: a systematic review. Minerva Urol Nefrol. (2018) 70:393–400. doi: 10.23736/S0393-2249.18.03113-2. 29856171

[20] Yang Q Chen HY Wang JN Han HQ Jiang L Wu WF. Alcohol promotes renal fibrosis by activating Nox2/4-mediated DNA methylation of Smad7. Clin Sci (Lond). (2020) 134:103–22. doi: 10.1042/CS20191047, PMID: 31898747

[21] Varga ZV Matyas C Paloczi J Pacher P. Alcohol misuse and kidney injury: epidemiological evidence and potential mechanisms. Alcohol Res. (2017) 38:283–8. 28988579 10.35946/arcr.v38.2.10 PMC5513691

[22] Yu JT Hu XW Chen HY Yang Q Li HD Dong YH. DNA methylation of FTO promotes renal inflammation by enhancing m(6)a of PPAR-alpha in alcohol-induced kidney injury. Pharmacol Res. (2021) 163:105286. doi: 10.1016/j.phrs.2020.105286, PMID: 33157234

