# Supplementary figures and images for: Association between alcohol consumption and kidney stones in American adults: 2007–2016 NHANES
Source: Front Public Health. 2023 Apr 14;11:1156097. doi: 10.3389/fpubh.2023.1156097 (PMC10140583; doi:10.3389/fpubh.2023.1156097)

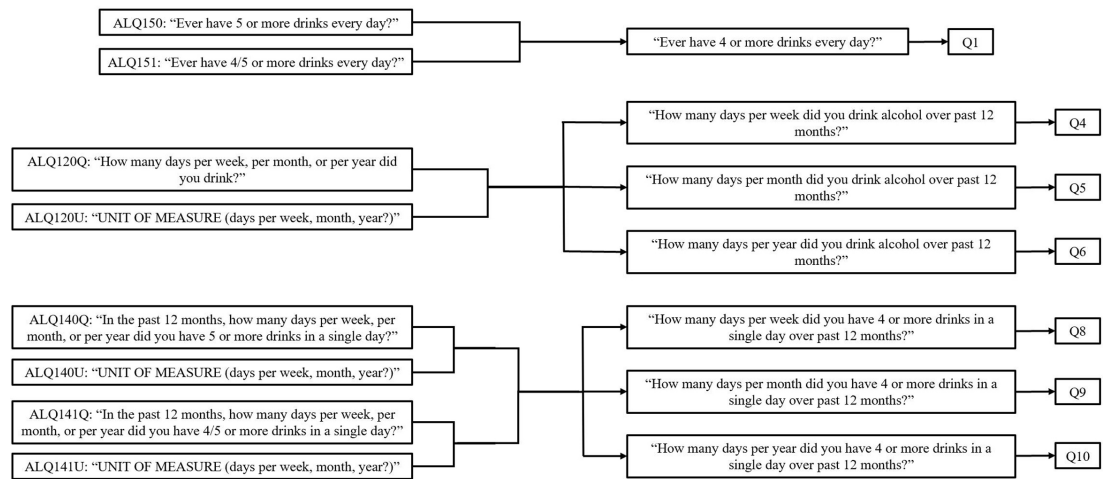

**Supplementary Figure 1.** Consolidation of questions

Supplement: Supplementary file 1 [file Presentation_1.PDF]
